# Uncovering the Repertoire of Endogenous Flaviviral Elements in Aedes Mosquito Genomes

**DOI:** 10.1128/JVI.00571-17

**Published:** 2017-07-12

**Authors:** Yasutsugu Suzuki, Lionel Frangeul, Laura B. Dickson, Hervé Blanc, Yann Verdier, Joelle Vinh, Louis Lambrechts, Maria-Carla Saleh

**Affiliations:** a Institut Pasteur, Viruses and RNA Interference Unit, CNRS Unité Mixte de Recherche 3569, Paris, France; b Institut Pasteur, Insect-Virus Interactions Group, Department of Genomes and Genetics, CNRS Unité de Recherche Associée 3012, Paris, France; c Spectrométrie de Masse Biologique et Protéomique, CNRS Unité de Service et de Recherche 3149, École Supérieure de Physique et de Chimie Industrielles Paris, Paris, France; University of Texas Southwestern Medical Center

**Keywords:** ancient flaviviral infections, endogenous viral elements, mosquito-borne viruses, mosquito genome, viral infectious diseases

## Abstract

Endogenous viral elements derived from nonretroviral RNA viruses have been described in various animal genomes. Whether they have a biological function, such as host immune protection against related viruses, is a field of intense study. Here, we investigated the repertoire of endogenous flaviviral elements (EFVEs) in Aedes mosquitoes, the vectors of arboviruses such as dengue and chikungunya viruses. Previous studies identified three EFVEs from Aedes albopictus cell lines and one from Aedes aegypti cell lines. However, an in-depth characterization of EFVEs in wild-type mosquito populations and individual mosquitoes *in vivo* has not been performed. We detected the full-length DNA sequence of the previously described EFVEs and their respective transcripts in several A. albopictus and A. aegypti populations from geographically distinct areas. However, EFVE-derived proteins were not detected by mass spectrometry. Using deep sequencing, we detected the production of PIWI-interacting RNA-like small RNAs, in an antisense orientation, targeting the EFVEs and their flanking regions *in vivo*. The EFVEs were integrated in repetitive regions of the mosquito genomes, and their flanking sequences varied among mosquito populations. We bioinformatically predicted several new EFVEs from a Vietnamese A. albopictus population and observed variation in the occurrence of those elements among mosquitoes. Phylogenetic analysis of an A. aegypti EFVE suggested that it integrated prior to the global expansion of the species and subsequently diverged among and within populations. The findings of this study together reveal the substantial structural and nucleotide diversity of flaviviral integrations in Aedes genomes. Unraveling this diversity will help to elucidate the potential biological function of these EFVEs.

**IMPORTANCE** Endogenous viral elements (EVEs) are whole or partial viral sequences integrated in host genomes. Interestingly, some EVEs have important functions for host fitness and antiviral defense. Because mosquitoes also have EVEs in their genomes, characterizing these EVEs is a prerequisite for their potential use to manipulate the mosquito antiviral response. In the study described here, we focused on EVEs related to the Flavivirus genus, to which dengue and Zika viruses belong, in individual Aedes mosquitoes from geographically distinct areas. We show the existence *in vivo* of flaviviral EVEs previously identified in mosquito cell lines, and we detected new ones. We show that EVEs have evolved differently in each mosquito population. They produce transcripts and small RNAs but not proteins, suggesting a function at the RNA level. Our study uncovers the diverse repertoire of flaviviral EVEs in Aedes mosquito populations and contributes to an understanding of their role in the host antiviral system.

## INTRODUCTION

Endogenous viral elements (EVEs), also known as viral fossils, are whole or partial viral sequences integrated in host genomes (1). When viral DNA integration occurs in the germ line, it can be inherited and retained in the host genome as evidence of ancient viral infections. Retrovirus-derived EVEs are the best-known examples since retroviruses actively integrate their DNA into the host genome as part of their life cycle during infection. However, single-stranded DNA virus-derived elements have been detected in plants (2), and more recently, non-retrovirus-derived EVEs have been shown in various animal hosts (3–7). Indeed, recent advances in bioinformatics have dramatically changed the landscape of paleovirology. *In silico* surveys were used to screen for EVEs in various animal genomes and identified a number of nonretroviral EVE sequences belonging to several virus families (5–8). The integrated viral elements mostly accumulate random mutations that render them inactive. In several instances, however, EVEs have maintained open reading frames (ORFs) and produce functional proteins that can serve during infections by closely related viruses (9–12). For example, endogenous bornavirus-like nucleoproteins from the thirteen-lined ground squirrel, Ictidomys tridecemlineatus (itEBLN), were the first nonretroviral EVEs demonstrated to serve as a negative regulator against infection by a related virus (10). Overexpression of itEBLN inhibits Borna disease virus (BDV) infection in mammalian cell lines, presumably by decreasing BDV polymerase activity. More recently, a virophage mavirus which is a parasite of the Cafeteria roenbergensis virus (CroV) was shown to be endogenized as an EVE in a marine flagellate, Cafeteria roenbergensis (9). CroV infection activates the endogenized mavirus genes in C. roenbergensis and produces infectious mavirus particles. These particles are secreted and protect surrounding flagellates from subsequent CroV infection. These studies demonstrate that EVEs can play critical roles in the host antiviral system.

Like most species examined, Aedes mosquitoes also have EVEs in their genomes. Aedes aegypti and Aedes albopictus mosquitoes are major vectors of arthropod-borne viruses (arboviruses), such as dengue virus (DENV) and Zika virus (ZIKV). DENV and ZIKV are members of the Flavivirus genus, which consists of enveloped viruses with a positive-sense, single-stranded RNA genome. In addition to these medically important mosquito-borne flaviviruses, Aedes mosquitoes in nature are infected by insect-specific flaviviruses (ISFs), such as cell-fusing agent virus (CFAV), Kamiti River virus (KRV), and Aedes flavivirus (AEFV) (13–15). Crochu et al. experimentally identified four endogenous flaviviral elements (EFVEs), three in A. albopictus cell lines and one in A. aegypti cell lines, which contain nonstructural (NS) genes related to ISFs (4). In the same study, a partial DNA sequence of these EFVEs was detected in A. albopictus and A. aegypti mosquitoes *in vivo* (4). Another study showed that it was possible to partially amplify, using degenerate primers, the NS5 region of flaviviruses in A. albopictus mosquitoes collected in northern Italy (16). In addition to these experimentally validated studies, *in silico* studies identified 28 and 24 genomic locations harboring EFVEs in A. aegypti and A. albopictus reference genomes, respectively (5, 8). All of the predicted EFVEs in A. aegypti mosquitoes were related to ISFs. For the 24 EFVEs predicted by Chen et al. in A. albopictus mosquitoes, they were mostly partial NS1 or NS5 sequences close to ISF and DENV sequences (8). However, no experimental validation of any of these bioinformatically predicted EFVEs has been performed either in vitro or *in vivo*.

To support our hypothesis that EFVEs could be used to manipulate the mosquito antiviral response to stop arbovirus transmission to the human host, we conducted a comprehensive characterization of EFVEs in Aedes mosquitoes representative of natural populations worldwide. We specifically focused on the only four EFVEs experimentally identified by Crochu et al. (4) and their known flanking regions. We investigated the EFVEs from several populations of each species sampled from geographically distinct locations. We show the presence of EFVE DNA and RNA transcripts *in vivo*. We confirmed the production of small RNAs derived from EFVE RNA *in vivo*. We further performed sequencing analysis of genomic DNA from A. albopictus mosquitoes and applied an *in silico* screening procedure that identified several new EFVEs in this species. Together, our results demonstrate the ubiquitous presence of diverse EFVEs *in vivo* and contribute to an understanding of the putative role of these elements in the antiviral defense system of mosquitoes during EFVE-related viral infection.

(This article was submitted to an online preprint archive [17].)

## RESULTS

### Detection of endogenous flaviviral elements in Aedes mosquitoes.

**FIG 1:**
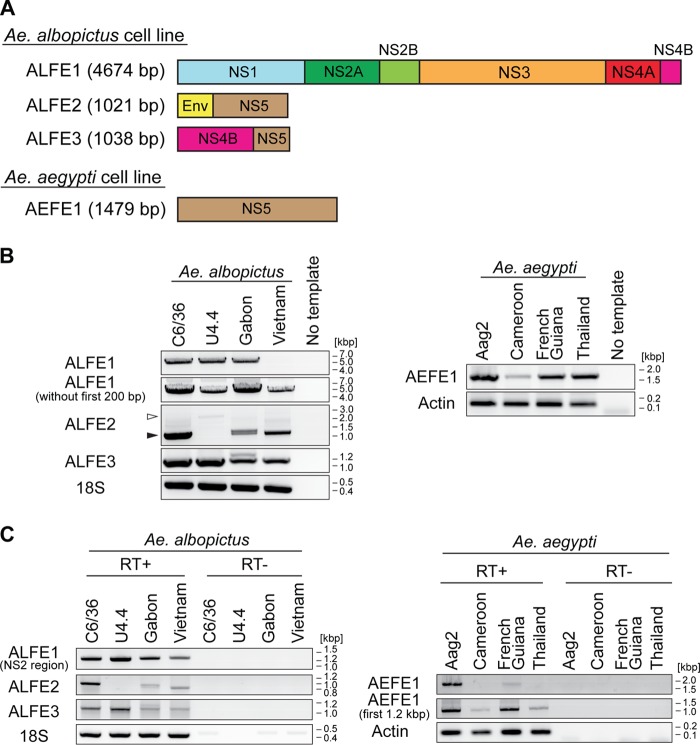
Detection of ALFE and AEFE DNA and RNA in Aedes mosquitoes. (A) Schematic representation of ALFE1 to ALFE3 and AEFE1. (B and C) Detection of ALFE1 to ALFE3 and AEFE1 DNA by PCR (B) and transcripts by RT-PCR (C) in C6/36 and U4.4 cell lines and the A. albopictus Gabon and Vietnam strains (left) and in the Aag2 cell line and the A. aegypti Cameroon, French Guiana, and Thailand strains (right). Black arrowheads in panel B, ALFE2-derived bands in the C6/36 cell line and in the Gabon and Vietnam strains; white arrowheads in panel B, ALFE2 fused with the host sequence in the U4.4 cell line. 18S rRNA and actin genes were used as controls for A. albopictus and A. aegypti, respectively.

Previous *in silico* studies predicted dozens of genomic locations harboring EFVEs in Aedes mosquito genomes (5, 8). However, only four annotated EFVEs with known flanking regions have been experimentally validated in vitro (by amplification of the full-length DNA sequence) and *in vivo* (by amplification of the partial DNA sequence) (4). We focused on these four annotated EFVEs that have been characterized for containing flaviviral nonstructural (NS) genes, three in A. albopictus (one named CSA1 and two unnamed) and one in A. aegypti (named CSA2) (4). For simplicity, we renamed these EFVEs A. albopictus flaviviral element (ALFE) 1 (ALFE1) to ALFE3 and A. aegypti flaviviral element (AEFE) 1 (AEFE1) (Fig. 1A). ALFE1 to ALFE3 were originally identified in the C6/36 cell line (A. albopictus), and AEFE1 was originally identified in the A20 cell line (A. aegypti). Laboratory and field-collected A. albopictus and A. aegypti mosquitoes were positive by PCR for the NS3 and/or NS5 region of both ALFEs and AEFE (4). However, the full-length elements were not confirmed in individual mosquitoes. Because geographical origin is expected to be associated with genetic divergence due to selective pressures particular to each geographical region and/or genetic drift, we explored EFVEs in mosquitoes from different parts of the world. We used A. albopictus mosquitoes from Gabon and Vietnam and A. aegypti mosquitoes from Cameroon, French Guiana, and Thailand. First, we attempted to detect full-length DNA for ALFE1 to ALFE3 and AEFE1 *in vivo*. Cell lines corresponding to each mosquito species (the Aag2 cell line for A. aegypti mosquitoes and the C6/36 and U4.4 cell lines for A. albopictus mosquitoes), in addition to individual mosquitoes, were used. PCR with primer pairs whose sequences started and ended at the very first and the very last nucleotide of each EFVE showed amplicons with the expected sizes for ALFE1 to ALFE3 and AEFE1, except for ALFE1 in the A. albopictus Vietnam strain (for which no amplification band was present) and ALFE2 in the U4.4 cell line (for which a larger band was present) (Fig. 1B, white arrowhead). We further characterized ALFE1 in the Vietnam strain by PCR with different primer pairs. ALFE1 was amplified with a primer pair targeting the element without the first 200 bp at its 5′ end (Fig. 1B). This result suggests that ALFE1 in A. albopictus Vietnam differs from ALFE1 in the A. albopictus Gabon strain and C6/36 and U4.4 cells in the first 200 bp of the element. For ALFE2, sequencing of the PCR product in U4.4 cells showed a partial ALFE2 sequence fused with unannotated mosquito sequences, suggesting that ALFE2 is recombined in U4.4 cells.

Next, we searched for the transcripts of ALFE1 to ALFE3 and AEFE1 by reverse transcriptase (RT) PCR. Previous work by Crochu et al. showed the ALFE1 transcript in C6/36 cells but not *in vivo*, while ALFE2, ALFE3, and AEFE1 mRNAs were not searched for in vitro or *in vivo* (4). The ALFE1 transcript was observed with a primer pair targeting the NS2 region but not a primer pair targeting the full-length element (Fig. 1C). ALFE2 and -3 transcripts were present in all samples, except for ALFE2 in U4.4 cells. The full-length AEFE1 transcript was observed in Aag2 cells and in the A. aegypti French Guiana strain but not in the A. aegypti Cameroon and Thailand strains. The first 1.2 kb of the AEFE1 transcripts was detected in all A. aegypti samples (Fig. 1C). The A. aegypti French Guiana strain was the only one to show the full-length transcript of AEFE1 among all A. aegypti strains tested. Altogether, our results indicate that ALFE1 to ALFE3 and AEFE1 were established by ancient viral infections in nature and not by artificial recombination in cell culture. In addition, ALFE1 to ALFE3 and AEFE1 are almost identical at the sequence level, and their transcripts are expressed among Aedes mosquito populations from different parts of the world.

### ALFEs and AEFEs produce piRNA-like molecules in Aedes mosquitoes.

**FIG 2:**
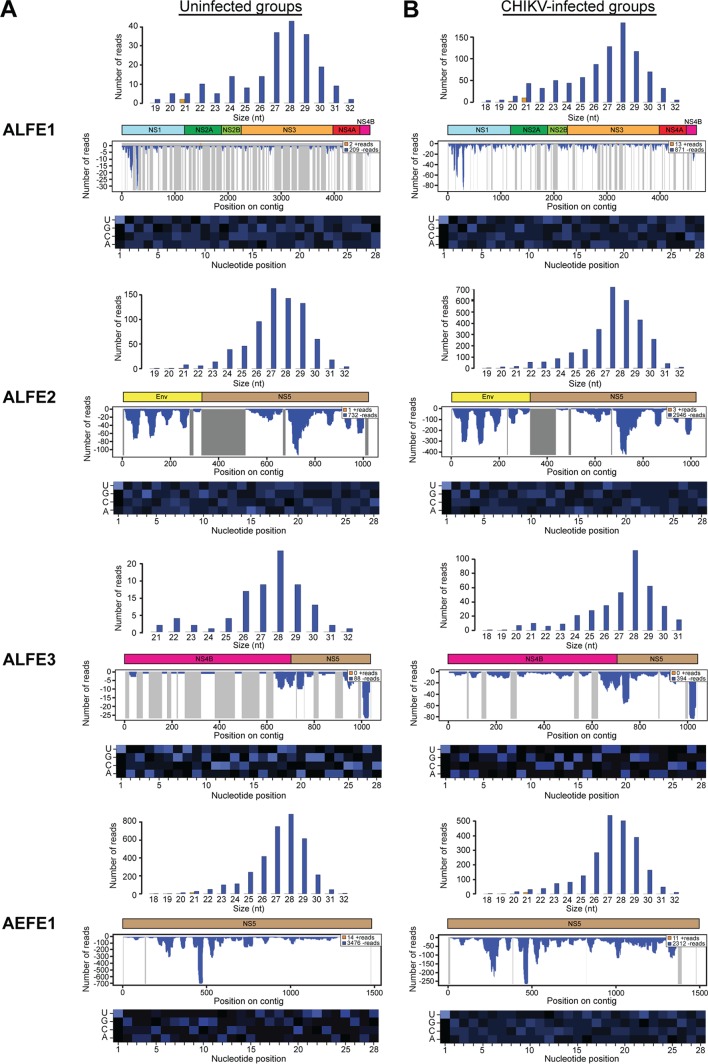
Primary piRNA-like small RNA production from ALFE1 to ALFE3 and AEFE1. The size distribution (top) and profiles (middle) of small RNAs mapped to ALFE1 to ALFE3 in A. albopictus mosquitoes and AEFE1 in A. aegypti mosquitoes not infected with CHIKV (A) or infected with CHIKV (B) are shown. Orange and blue bars, positive- and negative-stranded reads, respectively. For the small RNA profiles, the *x* axis represents the nucleotide position on the ALFE1-, ALFE2-, ALFE3-, or AEFE1-containing contig; the *y* axis shows the number of reads for each nucleotide position; gray lines, uncovered regions. (Bottom) Relative nucleotide frequency per position of the 28-nucleotide (nt) ALFE1-, ALFE2-, ALFE3-, or AEFE1-derived small RNAs shown as a heat map. The intensity varies in correlation with frequency.

**FIG 3:**
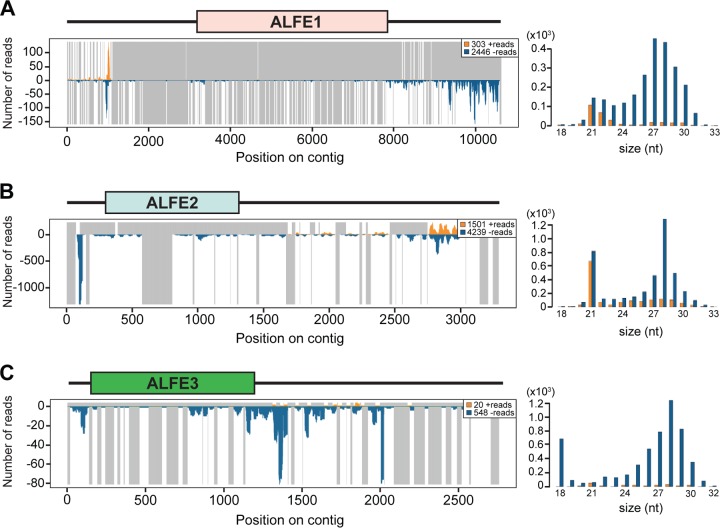
Small RNA production from ALFE1 to ALFE3 and their flanking regions in the A. albopictus genome. The small RNA profiles (left) and the size distributions (right) of small RNAs mapped to ALFE1 (A), ALFE2 (B), and ALFE3 (C) and their flanking sequences are shown. The schematic representation in each panel indicates the ALFE and its flanking region. The *x* axis represents the nucleotide position on the respective contig. The *y* axis shows the number of reads for each nucleotide position. Orange and blue bars, positive- and negative-stranded small RNAs, respectively; gray lines in the left panels, uncovered regions.

We previously reported that A. albopictus and A. aegypti infected with chikungunya virus (CHIKV) produce viral cDNA through endogenous retrotransposon activity and that this viral cDNA generates small interfering RNAs (siRNAs) and PIWI-interacting RNAs (piRNA) mediating viral persistence (18). CHIKV is an alphavirus from the Togaviridae family, which consists of enveloped viruses with a single-stranded, positive-sense RNA genome. CHIKV is also a mosquito-borne virus with a great impact on human health (19). To check if ALFE1 to ALFE3 and AEFE1 transcripts were also capable of generating small RNAs, such as CHIKV-derived viral cDNA, we reanalyzed small RNA libraries from A. albopictus and A. aegypti mosquitoes that were infected or uninfected with CHIKV and that were already available in the laboratory (Fig. 2). The majority of small RNAs that mapped to ALFE1 to ALFE3 and AEFE1 were 27 to 29 bases in length with an enrichment of uridine at the first position of the small RNA and were thus identified as primary piRNA-like molecules (Fig. 2A). We observed that piRNAs derived from these endogenous viral elements were only in the antisense orientation. CHIKV infection did not affect the size distribution and profiles of the small RNAs derived from ALFE1 to ALFE3 and AEFE1 (Fig. 2B). We examined the production of piRNAs targeting the flanking regions of ALFE1 to ALFE3 determined by Crochu et al. (4). We detected the production of only antisense-strand piRNA-like molecules on these regions, similar to the findings for ALFE1 to ALFE3 (Fig. 3). In addition, the flanking regions of ALFE1 and -2 produced 21-nucleotide-long small RNAs corresponding to siRNAs. These results indicate that ALFE1 to ALFE3 and AEFE1 produce piRNAs only in the antisense orientation and also that these elements are located in specific regions of the genome that generate long transcripts for endogenous siRNA and piRNA production.

### DENV infection does not affect AEFE1 transcript abundance in A. aegypti mosquitoes.

**FIG 4:**
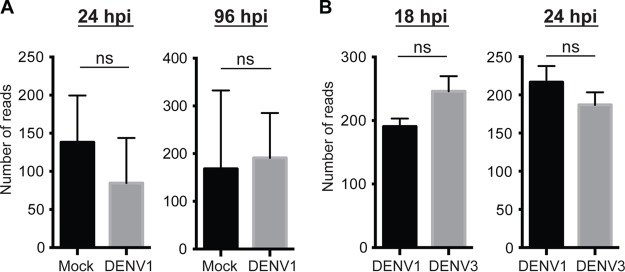
DENV1 and -3 infections have no detectable impact on AEFE1 transcript expression *in vivo*. The AEFE1 transcription level was analyzed by use of the DESeq2 package and a transcriptome data set from A. aegypti mosquitoes infected or not infected (Mock) with DENV1 (A) and A. aegypti mosquitoes infected with DENV1 or -3 (B) at the indicated time points. The average number of reads was calculated from several individual libraries: 7 and 17 libraries for mock- and DENV1-infected mosquitoes, respectively, at 24 h postinfection (hpi); 6 and 17 libraries for mock- and DENV1-infected mosquitoes infected, respectively, at 96 h postinfection; and 3 for each library for DENV1- or DENV3-infected mosquitoes at both time points. Statistical significance was determined by use of the DESeq2 package. ns, no significant difference (adjusted *P* value, >0.05).

Virus infections affect the expression of a number of host genes; for instance, DENV suppresses immune gene expression in the A. aegypti Aag2 cell line (20). The presence of transcripts from ALFEs and AEFEs in Aedes mosquitoes prompted us to check whether the expression level of their transcripts was altered during DENV infections *in vivo*. We utilized unpublished transcriptome data sets from female A. aegypti mosquitoes that were orally infected with either of two DENV serotypes (DENV serotype 1 [DENV1] and DENV3) or that were uninfected, which were available in the Lambrechts laboratory, to examine AEFE1 transcript expression. DENV1 infection did not affect AEFE1 transcript expression at 24 and 96 h postinfection (Fig. 4A). No significant difference in AEFE1 expression was observed between DENV1- and DENV3-infected A. aegypti mosquitoes at 18 and 24 h postinfection (Fig. 4B). The data suggest that AEFE1 is constitutively transcribed and not affected by DENV1 and -3 infections in adult female A. aegypti mosquitoes.

### Searching for new flavivirus-like elements in Aedes mosquitoes.

**FIG 5:**
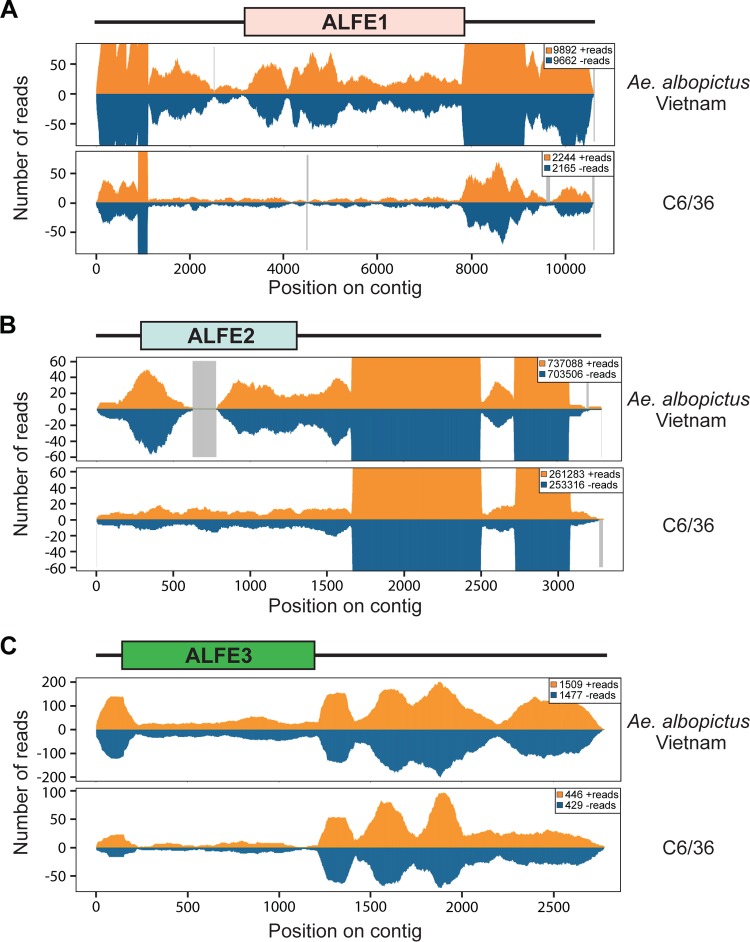
ALFEs are integrated into repetitive regions in Aedes mosquito genomes. DNA reads from the A. albopictus Vietnam strain and C6/36 cell DNA library were mapped to ALFE1 (A), ALFE2 (B), and ALFE3 (C) and their flanking regions. Orange and blue, positive- and negative-stranded reads, respectively. The box at the top of each panel indicates each ALFE, and the black bars represent the flanking regions. The *x* axis represents the nucleotide position on the ALFE-containing contig. The *y* axis shows the number of reads for each nucleotide position. Gray lines, uncovered regions.

PCR for full-length ALFE1 DNA in the A. albopictus Vietnam strain did not result in amplification (Fig. 1B). We then performed PCR with primer pairs targeting ALFE1 and its flanking regions and amplified bands with unexpected sizes (data not shown). Because of the heterogeneity of the PCR products, we moved on to confirm the existence of ALFE1 and to search for new ALFEs in A. albopictus. We performed whole-genome DNA sequencing of the A. albopictus Vietnam strain and the C6/36 cell line. First, we mapped reads from the A. albopictus Vietnam strain and C6/36 cell DNA libraries to the ALFE1 to ALFE3 sequences and to the sequences of their respective flanking regions (Fig. 5). DNA coverage showed the presence of full-length ALFE1 in the genomes of the Vietnam strain and of C6/36 cells (Fig. 5A), despite the lack of PCR amplification. In addition, the relative coverage of the flanking regions of the ALFE1 to ALFE3 sequences was substantially higher, indicating that these elements are integrated in multicopy regions (Fig. 5A to C). We also observed a similar trend of insertion of ALFEs in multicopy sequences when analyzing the genome sequence of the A. albopictus Foshan strain, available in the VectorBase database (data not shown).

**FIG 6:**
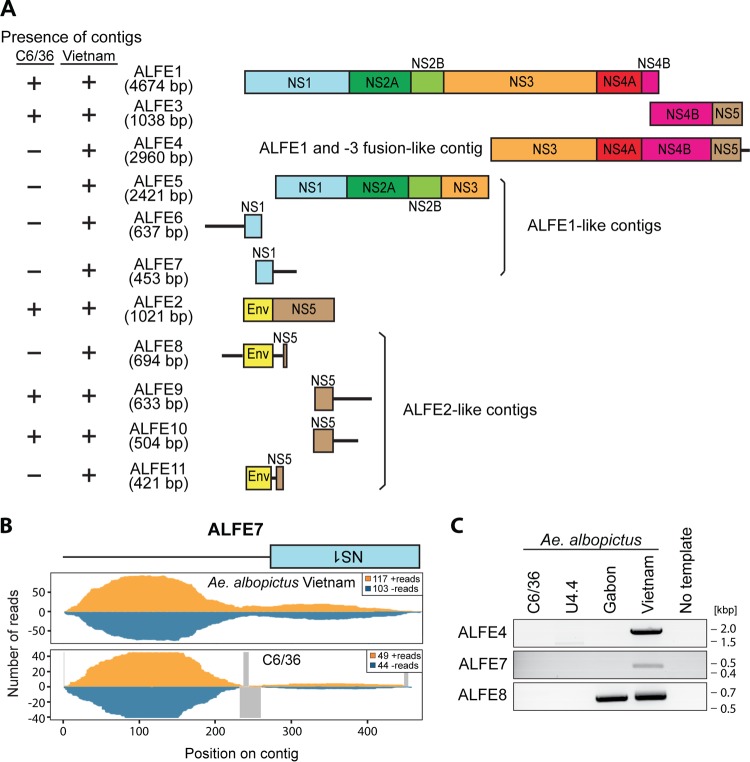
New ALFEs identified in the A. albopictus Vietnam strain. The *in silico* pipeline generated 8 new ALFE-like contigs. (A) Schematic representation of ALFE contigs. ALFE4 is a fusion element composed of ALFE1 and -3. ALFE5 to ALFE7 are partial sequences of ALFE1. ALFE8 to ALFE11 are ALFE2-like contigs. The boxes indicate ALFE-derived sequences, and the black lines indicate non-ALFE sequences. The presence (+) or absence (−) of ALFE-like contigs is summarized in the left column. (B) DNA coverage of ALFE7 with the A. albopictus Vietnam DNA library. Orange and blue, positive- and negative-stranded DNA, respectively; gray lines, uncovered regions. (C) ALFE4, -7, and -8 DNA detection in cells of the C6/36 and U4.4 cell lines and in the A. albopictus Gabon and Vietnam strains by PCR.

To identify new ALFEs, we performed an *in silico* screening based on an iterative mapping and assembly procedure using ALFE1 to ALFE3 sequences as scaffolds and the DNA library from the A. albopictus Vietnam strain as the query (details are provided in Materials and Methods). This *in silico* screening yielded eight contigs named ALFE4 to ALFE11 harboring partial sequences of ALFE1 to ALFE3 (Fig. 6A). For instance, ALFE4 was composed of a portion of ALFE1 followed by full-length ALFE3. We detected different genomic sequences flanking each ALFE, suggesting that multiple versions of each ALFE are present in the genome of the A. albopictus Vietnam strain. The sequences of the nearby regions are unique to Aedes mosquitoes, as a search for homology against the sequences in the GenBank and VectorBase databases did not show homology with any known organisms. Moreover, the six-phase translation of the flanking sequences did not show any similarity with known proteins from the UniProt Knowledge database.

**FIG 7:**
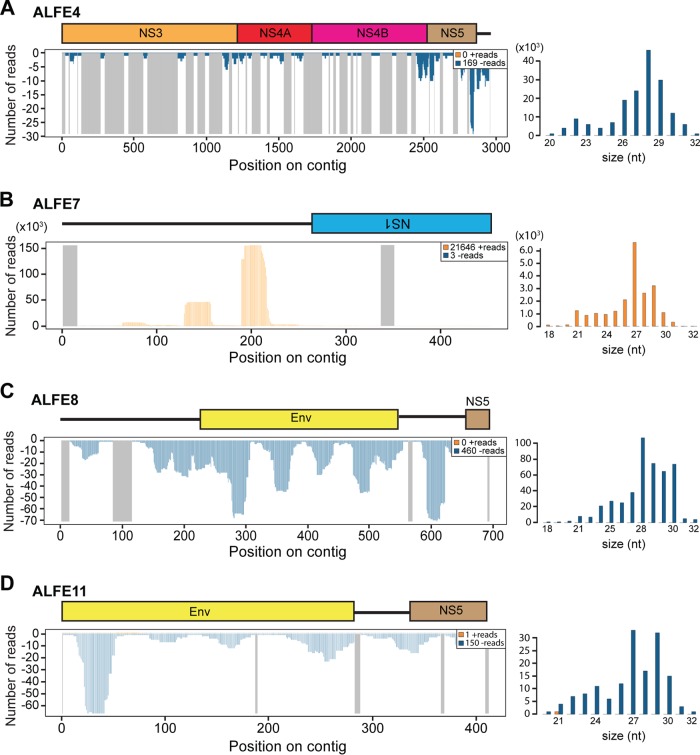
Small RNA production from ALFE4, -7, -8, and -11. The small RNA profiles (left) and size distribution (right) of small RNAs mapped to the ALFE4 (A), ALFE7 (B), ALFE8 (C), and ALFE11 (D) sequences are shown. The schematic representation in each panel indicates each ALFE and its flanking region. The *x* axis represents the nucleotide position on the contig. The *y* axis shows the number of reads for each nucleotide position. Orange and blue, positive- and negative-stranded small RNAs, respectively; gray lines in the left panels, uncovered regions.

To confirm the existence of the bioinformatically predicted ALFE4 to ALFE11, we used two different assessments: (i) the quality of the DNA mapping of the A. albopictus Vietnam strain and C6/36 cell reads to the new ALFE contigs and (ii) PCR with primer pairs specific to the new ALFE contigs. For DNA mapping, the depth and continuity of coverage along the ALFE sequences were used to confirm the existence of ALFEs in the Vietnam strain. Figure 6B shows the DNA coverage and the continuity of the reads on ALFE7 as an example of the validation. In the A. albopictus Vietnam strain, the reads continuously covered the ALFE7 contig sequence, while in C6/36 cells, there were differences in the continuity of coverage (a gap was present). In addition, the read counts corresponding to the flanking regions of ALFE7 confirmed that this element is integrated in multicopy sequences in the A. albopictus genome. All the predicted ALFEs were confirmed by PCR amplification to be present in the Vietnam strain, except for ALFE5, which could not be distinguished from ALFE1 due to sequence similarity. Figure 6C shows an example of the PCR amplification products of the predicted ALFE4, -7, and -8. These three elements were detected in the A. albopictus Vietnam strain, whereas only ALFE8 was present in the Gabon strain. Neither C6/36 cell nor U4.4 cell genomes showed the presence of these elements. In addition, we checked for small RNA production from the newly described ALFEs. As observed with ALFE1 to ALFE3, the bioinformatically predicted ALFEs and their flanking sequences were covered only by antisense piRNA-like molecules with a U-1 bias (Fig. 7).

**FIG 8:**
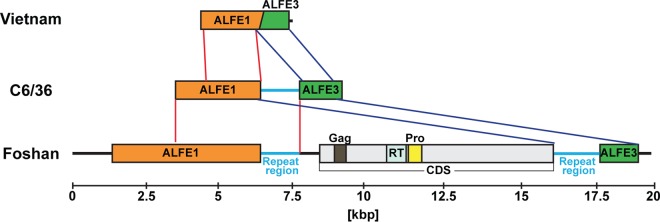
Comparison of the ALFE1/ALFE3 sequences in the A. albopictus Vietnam strain, Foshan strain, and C6/36 cells. Schematic representations of ALFE4 (from the Vietnam strain), an ALFE4-like element (from C6/36 cells), and Foshan strain ALFE1 and -3 with their flanking regions are shown. ALFE1/ALFE3 in the Foshan strain has a gap of approximately 11 kbp in length with a CDS harboring retrotransposon Gag, reverse transcriptase, and protease domains and repeat sequences at both the 5′ and 3′ extremities. Overlapped regions of ALFE1 and -3 are visualized with red and blue lines, respectively. Light blue bars, repeat sequences.

Lastly, we compared the ALFE contigs identified from C6/36 cells and the A. albopictus Vietnam strain with A. albopictus Foshan genomic DNA supercontigs. We observed considerable variation in the flanking regions between the ALFEs in the different strains. For example, the *in silico* pipeline predicted the existence of ALFE4 (a fusion between ALFE1 and ALFE3) in the Vietnam strain (Fig. 8). When the ALFE4 contig was compared to the ALFE4-like contig in C6/36 cells, a 1.3-kbp host sequence was present between ALFE1 and -3. This host sequence also exists in the Foshan genome but is much longer (11 kbp) and contains a repeat sequence at both extremities of a coding DNA sequence (CDS) with Gag-, reverse transcriptase-, and proteinase-like domains. The analysis suggests that ALFE1 and -3 were originally the same element in the Vietnam strain (ALFE4) and were separated by insertion of a retrotransposon in the Foshan strain or vice versa.

### ALFE- and AEFE-derived proteins are undetectable by MS.

Because ALFE1 to ALFE3 and AEFE1 generated transcripts in vitro and *in vivo*, we checked whether they could produce detectable proteins. As antibodies against ALFEs, AEFEs, or similar flaviviruses are not available, we performed mass spectrometry (MS) analysis with the C6/36 and Aag2 cell lines to find ALFE- and AEFE-derived peptides. Proteins from C6/36 and Aag2 cells were purified and subjected to MS for bottom-up proteomics analysis. Although we identified Ago2, Dcr2, and Piwi5, as well as thousands of proteins from all the subcellular fractions ranging from 5.6 to 811 kDa in molecular mass (Table 1; see also Table S1 in the supplemental material), no ALFE- or AEFE-proteolytic peptides were identified in either C6/36 or Aag2 cells. Of note, it is not possible to use the expression level of the identified proteins to estimate a maximum expression level of ALFEs and AEFEs, because the ionization properties of proteotypic peptides can be very different. However, the number of identified proteins, their large molecular mass range, and their subcellular origin suggest that a large part of the proteome has been covered. Our result suggests that ALFE1 to ALFE3 and AEFE1 are not translated at a high level, if they are translated at all, in both mosquito cell lines.

**TABLE 1:** Proteomic characterization of C6/36 and Aag2 cell lines

Cell line	No. of times that proteolytic peptides were found^*a*^				No. of identified proteins^*b*^
	EFVEs	Piwi5	Dcr2	Ago2	
C6/36	0	3	0^*c*^	2	1,942 ± 637
Aag2	0	3	2	2	2,054 ± 291

a Number of times that proteolytic peptides corresponding to EVE proteins and positive controls (out of 3 experiments) were found.

b The average total number of identified proteins is depicted.

c C6/36 cells are Dcr2 deficient.

### Phylogenetic analysis of AEFE1 in A. aegypti mosquitoes.

**FIG 9:**
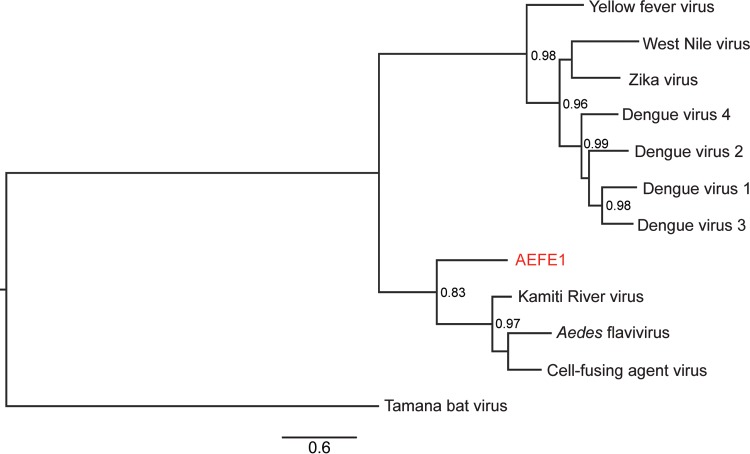
Phylogenetic relationships among ALFE1 and insect-specific flaviviruses. A maximum likelihood tree was generated using the Fast likelihood-based approach. ALFE1 DNA from individual mosquitoes of the A. aegypti Cameroon, French Guiana, and Thailand strains was sequenced. The tree was generated with Aedes flavivirus (GenBank accession no. AB488408.1), cell-fusing agent virus (GenBank accession no. NC_001564.2) and Kamiti River virus (GenBank accession no. AY149905.1) as ISFs and DENV1 (GenBank accession no. KX225493.1), DENV2 (GenBank accession no. AY858035.2), DENV3 (GenBank accession no. AY858047.2), DENV4 (GenBank accession no. KU523872.1), ZIKV (GenBank accession no. KU955595.1), West Nile virus (GenBank accession no. M12294.2), Yellow fever virus (GenBank accession no. NC_002031.1), and Tamana bat virus (GenBank accession no. AF285080.1) and rooted using Tamana bat virus. The scale bar indicates the number of nucleotide substitutions per site. Node values represent Shimodaira-Hasegawa (SH)-like branch support (only values of >0.7 are shown).

**FIG 10:**
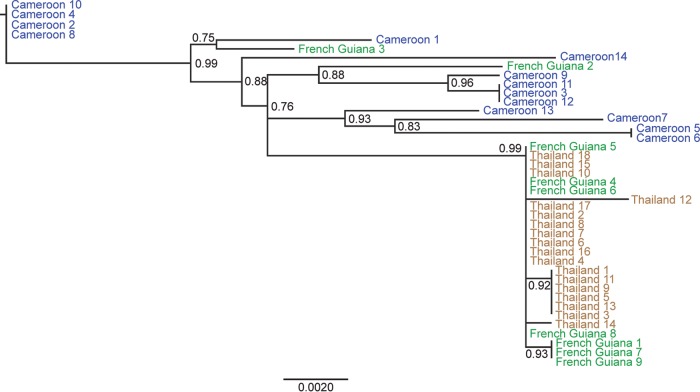
Phylogenetic relationships among AEFE1 sequences from different A. aegypti populations. A maximum likelihood tree was generated using the Fast likelihood-based approach. AEFE1 DNA from individual mosquitoes of the A. aegypti Cameroon, French Guiana, and Thailand strains was sequenced. The scale bar indicates the number of nucleotide substitutions per site, and node support values are shown for major nodes. The tree was rooted with ISF sequences (Fig. 9), but the outgroup was omitted for visual clarity. Node values represent Shimodaira-Hasegawa (SH)-like branch support (only values of >0.7 are shown).

To study the evolutionary history of AEFE1, we examined its genetic diversity in A. aegypti mosquitoes from Cameroon, French Guiana, and Thailand. The full-length sequence of AEFE1 in individual mosquitoes from each country was amplified and sequenced. Some individual mosquitoes from the Cameroon and Thailand populations did not show amplification of AEFE1. Next, we generated maximum likelihood phylogenetic trees of AEFE1 consensus sequences from each individual and their homologous sequences in the genomes of ISFs closely related to AEFE1, such as CFAV, KRV, and AEFV, as well as homologous sequences in related medically important flaviviruses. As expected, the AEFE1 sequence was closely related to the ISF sequences (Fig. 9). The phylogenetic analysis showed that among the three ISFs considered, KRV is the closest to AEFE1. To further examine the relationship among AEFE1 sequences from different geographical locations, an additional tree was generated and rooted with the ISFs as the outgroup but is presented without the ISFs for visual clarity (Fig. 10). The phylogenetic relationships among AEFE1 sequences mainly recapitulated the recent evolutionary history of A. aegypti populations. With two exceptions, the AEFE1 sequences in mosquitoes from the non-African (Thailand and French Guiana) populations were derived from AEFE1 sequences found in the African (Cameroon) population. A basal evolutionary position of African populations is typically observed for A. aegypti genes (21), consistent with the recent out-of-Africa geographical expansion of the species. Thus, the phylogenetic analysis suggests that AEFE1 evolved consistently with other host genes at the species level.

## DISCUSSION

Recently, reactivation of EVEs due to related or unrelated viral infections has been reported, strongly suggesting a role of EVEs during the immune response of the host (9–11). Dozens of EVEs comprising flavivirus-, rhabdovirus-, and reovirus-related EVEs have been bioinformatically predicted for different mosquito strains, and some of them were confirmed in mosquito cell lines or strains (4, 5, 7, 8, 22). However, few studies have been conducted to characterize EVEs *in vivo*, a necessary step to further investigate their role during viral infection of mosquitoes. Due to the current disease outbreaks caused by mosquito-borne flaviviruses, such as Zika and dengue viruses, we decided to study and characterize *in vivo* endogenous flaviviral elements (EFVEs) that were previously identified in A. albopictus and A. aegypti mosquito cell lines. To improve our understanding of the forces shaping the evolution of these EFVEs, we assessed their presence, transcription, small RNA production, protein production, and phylogeny using African, Asian, and South American populations of Aedes mosquitoes. These wild-type populations are epidemiologically relevant because they occur in regions where they act as the main arbovirus vectors (23, 24). To simplify the nomenclature, we propose to name the EFVEs identified in A. albopictus mosquitoes ALFE*x* and the ones identified in A. aegypti mosquitoes AEFE*x*, where *x* is a number.

We first showed the presence of full-length ALFE1 to ALFE3 DNA and AEFE1 DNA in several populations of individual A. albopictus and A. aegypti mosquitoes, respectively. This indicates that ALFE1 to ALFE3 and AEFE1 are likely derived from ancient events of flavivirus DNA integration in nature that have persisted during the course of evolution. We also detected complete or partial RNA transcripts of ALFE1 to ALFE3 and AEFE1 *in vivo*. The expression level of AEFE1 remained unchanged following DENV1 and -3 infection of A. aegypti mosquitoes, suggesting a constitutive expression. However, experiments addressing AEFE and ALFE transcriptional regulation during different virus infections or different abiotic or biotic stimuli should be designed and performed in the future.

In most documented cases, functional EVEs play a role at the protein level (9–12). We performed a powerful bottom-up proteomic approach to search for ALFE- and AEFE-proteolytic peptides in the C6/36 and Aag2 cell lines. The mass spectrometry analysis could not validate the expression of ALFE1 to ALFE3 and AEFE1 proteins in any of the subcellular fractions analyzed, while control peptides for each cell type were readily detected. Since ALFEs and AEFEs have been maintained in the cell lines and individual mosquitoes at the DNA and RNA levels, this result strongly suggests that ALFE and AEFE transcripts are not translated into proteins. However, EFVEs could need specific conditions to be translated. For instance, the mosquito cell lines that we used were established from larvae or embryos (25, 26). ALFEs and AEFEs could be translated only in specific tissues or stages of development *in vivo*. Another interesting possibility is that because the transcripts were observed by RT-PCR and RNA sequencing, ALFEs and AEFEs could have a function at the RNA level.

Small RNAs have critical roles in various aspects of host fitness and antiviral immunity in mosquitoes (18, 27–33). The piRNA pathway is one of the small RNA pathways and mainly controls nonself sequences, such as retrotransposable elements and DNA transposons (34–36). According to the PIWI proteins involved and available for piRNA biogenesis, they are classified into primary piRNAs or secondary piRNAs, with the latter being produced from an amplification cycle known as the ping-pong cycle using primary piRNAs as the template. Infections with arboviruses, such as CHIKV and DENV, have been shown to produce both primary and secondary piRNAs predominantly in the sense orientation in mosquitoes (18, 29, 37). We found that ALFE1 to ALFE3, AEFE1, and their flanking regions produced a population of primary piRNA-like molecules only in the antisense orientation and that these molecules were not affected during CHIKV infection. A recent study also found that some annotated genes, including EVEs, produce antisense primary piRNA-like small RNAs in A. aegypti mosquitoes and Aag2 cells (38, 39). Two nonexclusive hypotheses could account for the production of only primary piRNA-like molecules by ALFEs and AEFEs. First, the production of ALFE- and AEFE-derived primary piRNAs may occur only in specific tissues lacking PIWI proteins, such as Piwi5 and/or -6, involved in ping-pong amplification. Second, the transcript that produces primary piRNAs of EFVEs is predominantly transcribed in the antisense orientation. Indeed, we observed a similar tendency in piRNA production from the flanking regions of ALFEs and AEFEs, suggesting that primary piRNAs from EFVEs are produced from the same precursor transcript as their flanking regions. Curiously, in vertebrates, some murine EBLNs and their flanking regions also generate antisense piRNA-like small RNAs (40). This suggests that EVEs have common features in small RNA production across species and kingdoms. To elucidate how and why this is happening deserves further studies.

Regarding the function of these EFVE-derived piRNAs, it is tempting to propose that they could regulate infections by closely related viruses by targeting viral RNA in Aedes mosquitoes. ALFEs and AEFEs do not contain sequences of 24 to 30 nucleotides that perfectly span AEFV and KRV sequences, which are the most closely related known ISFs. However, piRNAs allow some mismatches with target sequences (41–43). If ALFE- and AEFE-derived piRNAs are loaded into the ping-pong amplification cycle during viral infection, a number of piRNAs that match the virus sequence could be produced and those piRNAs could contribute to the control of viral replication. We and others previously demonstrated that viral piRNAs (vpiRNAs) were detected in Aedes mosquitoes and in mosquito cell lines infected with mosquito-borne viruses, such as CHIKV and Rift Valley Fever virus (phlebovirus, Bunyaviridae family) (18, 30). Although the direct effects of vpiRNA on viral replication remain unclear, vpiRNAs could render the mosquitoes tolerant to infections with the viruses (18). By providing closely related piRNAs to the ping-pong cycle, ALFEs and AEFEs may contribute to the control of pathogenesis during exogenous viral infections.

Using a bioinformatics approach, we identified several new ALFEs composed of partial or complete ALFE1 to ALFE3 sequences in A. albopictus mosquitoes from Vietnam. Some of these new ALFEs were predicted only in our mosquito strain and were not found in the C6/36 cell line genome. PCR also showed variability in the detection of ALFE4 to ALFE11 among the mosquito strains, suggesting that the genome of each mosquito population has a different set of EFVEs. The recent genome sequencing of the A. albopictus Foshan strain by Chen et al. (8) found more than 20 EFVEs that were mostly related to NS1 or NS5 genes from ISFs. Likewise, we also found three elements containing NS1 and four containing NS5 out of eight identified ALFE-like contigs. Another study performed on field-collected A. albopictus mosquitoes in northern Italy detected flaviviral NS5 related to CFAV and KRV NS5 (16). Therefore, Aedes mosquitoes seem to have preferentially accumulated ancient flaviviral NS5 sequences in their genomes. There are two potential and not exclusive explanations for this: (i) NS5 could be preferably endogenized over other flaviviral sequences, and (ii) NS5 sequences could be positively maintained over generations. Although the mechanism of endogenization has not been elucidated, we and others have suggested that it happens in a retrotransposon-dependent manner (18, 44, 45). The genomes of A. albopictus and A. aegypti mosquitoes harbor 50% to 70% repetitive sequences, such as retrotransposons and long interspersed nuclear elements (LINEs) (8, 46). Accordingly, it is interesting to propose that the sequence of NS5 could be efficiently recognized by retrotransposons in Aedes mosquito genomes.

One interesting observation arose when we compared the newly identified ALFE4 from our Vietnam strain and ALFE4 from the Foshan strain of A. albopictus. In the Vietnam strain, ALFE4 appears as a fusion of ALFE1 and ALFE3. In the Foshan strain, a large CDS containing a retrotransposon RT-like domain with terminal repeat sequences exists between ALFE1 and ALFE3. Katzourakis and Gifford (5) observed in A. aegypti mosquitoes the presence of an almost entire flaviviral genome fragmented in several pieces across the mosquito genome. We propose that ALFE- and AEFE-like sequences are likely inserted in repetitive regions of the mosquito genome, where transposons can act as a trap for nonretroviral DNA. These intergenic flanking regions display substantial structural variation among mosquito populations, arguing for an active rearrangement and continuous change of EFVE-containing regions. Nevertheless, EFVE sequences have been maintained in each mosquito population, suggesting that these captured viral sequences play a role during host evolution.

It is, however, important to stress that we faced significant challenges working with the genome sequence of the A. albopictus Vietnam strain. This was presumably due to the difficulties in assembling the different contigs, despite a comfortable depth of coverage, due to the high content of repetitive sequences. Indeed, the currently available genome assemblies of A. albopictus and A. aegypti in the VectorBase database consist of thousands of unassembled supercontigs. There is an urgent need to assemble these fragmented reference genome sequences into end-to-end chromosome maps. It is hoped that the advent of new tools, such as long-read sequencing technologies and chromosome confirmation capture, will produce a leap forward in the study of mosquito genomes.

Finally, a phylogenetic analysis of AEFE1 in A. aegypti mosquitoes from Cameroon, French Guiana, and Thailand showed that the evolutionary history of AEFE1 was similar to that of most nuclear genes. Note that we did not investigate the phylogenetic relationships of ALFEs because the current lack of population genomics studies of A. albopictus mosquitoes would have made any interpretation difficult. According to population genomics studies of A. aegypti, this species originated in Africa and colonized the rest of the world following a single migration event, probably by traveling in ships along trading routes in past centuries (21). AEFE1 was genetically more diverse in the Cameroon strain than the French Guiana and Thailand strains, which is also consistent with the patterns of genetic diversity for nuclear genes. Our phylogenetic analysis suggests that the AEFE1 integration events occurred before A. aegypti mosquitoes expanded out of the African continent. However, the findings for two individual mosquitoes from French Guiana were inconsistent with the out-of-Africa model. This could be due to introgression from African mosquitoes subsequently introduced into South America (47) or because the sequences were not orthologous. We also found some A. aegypti individuals from Cameroon and Thailand in which the full-length AEFE1 could not be amplified, suggesting that both structural and nucleotide variants of AEFE1 exist within the same population. It is likely that following the initial integration event, complex evolutionary forces have shaped the genetic diversity of EFVEs that is observed today. Unraveling this diversity will be necessary to elucidate their potential functional role.

## MATERIALS AND METHODS

### Ethics statement.

The Institut Pasteur animal facility has received accreditation from the French Ministry of Agriculture to perform experiments on live animals in compliance with French and European regulations on the care and protection of laboratory animals. The rabbit blood draws performed during the course of this study were approved by the Institutional Animal Care and Use Committee at the Institut Pasteur under protocol number 2015-0032, in accordance with European directive 2010/63/UE and French legislation.

### Cell culture.

Cells of the C6/36 (ATCC CRL-1660) and U4.4 cell lines (A. albopictus) (26) and the Aag2 cell line (A. aegypti) (48) (kindly provided by G. P. Pijlman, Wageningen University, the Netherlands) were maintained at 28°C in L-15 Leibovitz medium (Gibco) supplemented with 10% fetal bovine serum (FBS; Gibco), 1% nonessential amino acids (Gibco), 2% tryptose phosphate broth (Sigma), and 1% penicillin-streptomycin (Gibco).

### Mosquitoes.

Laboratory colonies of A. aegypti mosquitoes were established from field collections in Cameroon (2014), French Guiana (2015), and Thailand (2013). Laboratory colonies of A. albopictus were established from field collections in Gabon (2014) and Vietnam (2011). All the experiments were performed within 16 generations of laboratory colonization. The insectary conditions for mosquito maintenance were 28°C, 70% relative humidity, and a 12-h light and 12-h dark cycle. Adults were maintained with permanent access to 10% sucrose solution. Adult females were offered commercial rabbit blood (BCL) twice a week through a membrane feeding system (Hemotek Ltd.).

### Experimental DENV infections *in vivo*.

Wild-type, low-passage-number DENV isolates (DENV1 KDH0030A [49], DENV3 GabonMDA2010 [50]) were originally obtained in 2010 from the serum of dengue patients. Informed consent of the patients was not necessary because the viruses isolated in cell culture are no longer considered human samples. KDH0030A was isolated at the Armed Forces Research Institute of Medical Sciences, Bangkok, Thailand. GabonMDA2010 was isolated at the Centre International de Recherches Médicales de Franceville, Gabon. Virus stocks were prepared and experimental mosquito infections were conducted as previously described (51). Briefly, 4- to 7-day-old female mosquitoes were deprived of sucrose for 24 h and transferred to a biosafety level 3 insectary. Washed rabbit erythrocytes resuspended in phosphate-buffered saline were mixed 2:1 with prediluted viral stock and supplemented with 10 mM ATP (Sigma-Aldrich). The viral stock was prediluted in Leibovitz L-15 medium with 10% FBS, 0.1% penicillin-streptomycin, and 1% sodium bicarbonate (Gibco) to reach an infectious titer ranging from 5 × 10^6^ to 1.1 × 10^7^ focus-forming units per ml of blood using a standard focus-forming assay in C6/36 cells (51). A control blood meal was prepared identically except that the supernatant of mock-inoculated cells replaced the viral suspension. Mosquitoes were offered the infectious or control blood meal for 30 min through a membrane feeding system (Hemotek Ltd.) set at 37°C with a piece of desalted pig intestine as the membrane. Following the blood meal, fully engorged females were selected and incubated at 28°C and 70% relative humidity under a 12-h light and 12-h dark cycle with permanent access to 10% sucrose.

### PCR and RT-PCR.

Total DNA was extracted from the mosquito cell lines or individual mosquitoes with a NucleoSpin tissue kit (Macherey-Nagel). Total RNA was extracted from the mosquito samples with the TRIzol reagent (Invitrogen). Following DNase I (Promega) treatment, cDNA was synthesized with Maxima H Minus reverse transcriptase and oligo(dT) primers according to the manufacturer's instructions (Thermo Fisher Scientific). PCR and nested PCR were performed with Dream*Taq* DNA polymerase (Thermo Fisher Scientific). The sequences of the primers are provided in Table 2.

**TABLE 2:** Primer pairs for PCR and RT-PCR

Target	Name of primer	Sequence
ALFE1 flanking region	ALFE1_-100F	CCCTGTCACACAAGCTTGGAG
	ALFE1_ + 99R	CCTACCATGGAGGGTGTTCTGTTTG
Full-length ALFE1	ALFE1_F	ATGGTGGTCGTCTTCACTATGTACATAC
	ALFE1_R	CTAATTCTTGCTGCAAGCTGAGTTCTG
ALFE1 without first 200 bp	ALFE1_200	GCACAAATAAACCATGCCTAATCTGCG
	ALFE1_R	CTAATTCTTGCTGCAAGCTGAGTTCTG
ALFE1 NS2 region	ALFE1_NS2_F	AGATCGGATTTTCACCCAGCGAAC
	ALFE1_NS2_R	TCTACAATGCTCGTTAAGTTTTAAGCG
Full-length ALFE2	ALFE2_F	ACGGCAGTAATGACCTGTGCTG
	ALFE2_R	CCCCAATACTTTATCTCCGTGCCG
Full-length ALFE3	ALFE3_F	CGAGATCACCGCGCGAATCC
	ALFE3_R	GTGGCTAGATTGTAGCCGCGAG
Full-length AEFE1	AEFE1_F	TCTGCCAGGGACGTGTACATG
	AEFE1_R	GTTTTCGTTGTTTGGTGTGATGGATGG
First 1.2 kbp of AEFE1	AEFE1_F	TCTGCCAGGGACGTGTACATG
	AEFE1_1200_R	GAAAGTGAAGGGCTACTCGAAGCTG
18S rRNA	18S_F	GGTCGGCGCGGTCGTAGTGTGG
	18S_R	TCCTGGT GGTGCCCTTCCGTCAAT
Actin	Actin_F	AAGGCTAACCGYGAGAAGATGAC
	Actin_R	GATTGGGACAGTGTGGGAGAC
ALFE4	ALFE_NS4_F	TTGTCCATTGAGATGTATATCAATACCCG
	ALFE3_R	GTGGCTAGATTGTAGCCGCGAG
ALFE6	ALFE6_F	AAAGCTTTACTACTCGAAAACTCCC
	ALFE7_R	TCCTTTATGTACAGATTTGTGAATGCG
ALFE7	ALFE7_F	TCACTCCGTGGAGCTGGATG
	ALFE7_R	TCTTTCTTAAAATTGATACCTTCCATTGATC
ALFE8	ALFE8_F	CTAAAACATTATGAGTGAAAATGGCAG
	ALFE8_R	GCTCTTCCTCATCTTGCAAGTCG
ALFE9	ALFE9_F	AGATGAGAACCTTGAAGACACCCTTAG
	ALFE9_R	CAGTCTGTGGTAATGTTGTTGGCG
ALFE10	ALFE10_F	AGATGAGAACCTTGAAGACACCC
	ALFE10_R	CAAATCACCAGCGAAGGCTTTC

### Transcriptome data analysis.

Individual midgut libraries of A. aegypti mosquitoes infected with DENV1 or DENV3 were prepared from total RNA extracts from individual midguts after quality control with a Bioanalyzer RNA 6000 kit (Agilent). Purification and fragmentation of mRNA, cDNA synthesis, end repair, A tailing, Illumina index ligation, and PCR amplification were performed using a TruSeq RNA sample preparation (v2; Illumina), followed by a cDNA quality check by use of a Bioanalyzer DNA 1000 kit (Agilent). Libraries were diluted to 10 pM after Qubit quantification (Thermo Fisher Scientific), loaded onto a flow cell, and clustered with TruSeq SR Cluster kit v3-HS on a cBot system (Illumina). Single-end reads of 51 nucleotides in length were generated on a HiSeq2000 sequencing platform (Illumina). Sequencing reads with a quality score of <30 were trimmed using the Cutadapt program (https://cutadapt.readthedocs.io/en/stable/). Passing-filter reads were mapped to A. aegypti transcripts (AaegL3.1; http://vectorbase.org) using the Bowtie 2 tool and then processed with the SAMtools suite to create a matrix of raw counts used for gene expression analysis by the DESeq2 package.

### Small RNA and genome DNA libraries.

To analyze small RNA production from ALFEs and AEFEs, we used small RNA libraries of A. albopictus and A. aegypti mosquitoes infected or not infected with CHIKV that are publically available in the Sequence Read Archive under accession number SRP062828. For genomic DNA libraries, total DNA was extracted from C6/36 cells and the A. albopictus Vietnam strain using a NucleoSpin tissue kit (Macherey-Nagel). Genomic DNA was then sheared into 200-bp fragments using a Covaris S220 device with the following parameters: peak incident power, 175; duty factor, 10; cycle burst, 200; and duration, 180 s. Genomic DNA libraries were prepared using a Kapa LTP library preparation kit Illumina Platforms (Kapa Biosystems). The library was amplified with 10 PCR cycles, and 2 × 151 paired-end reads were sequenced on a NextSeq 500 sequencer.

For bioinformatics analysis, the quality of the fastq files was assessed with FastQC software (www.bioinformatics.babraham.ac.uk/projects/fastqc/). Low-quality bases and adaptors were trimmed from each read by use of the Cutadapt program. Only reads with an acceptable quality (Phred score, 20) were retained. A second set of graphics was generated by the FastQC software using the fastq files trimmed by the Cutadapt program. Reads were mapped to target sequences using the Bowtie 1 tool with the −v 1 (one mismatch between the read and its target) or the Bowtie 2 tool with default options for the small RNA or DNA library, respectively. The Bowtie 1 tool (small RNA library) and the Bowtie 2 tool (DNA library) generate results in sequence alignment/map (SAM) format. All SAM files were analyzed by the SAMtools package to produce bam indexed files. Homemade R scripts with Rsamtools and Shortreads in Bioconductor software were used for analysis of the bam files.

### *In silico* screening for new EFVEs.

To identify all versions of EFVEs in A. albopictus mosquito DNA contigs, we developed an iterative bioinformatics pipeline. Each iteration is composed of 4 steps: (i) BLASTN analysis using raw reads from the DNA library as the database and an E value threshold of 1E−20, (ii) read extraction according to the BLASTN result, (iii) assembly of these reads using the SPAdes (v3) genome assembler, and (iv) extraction of contigs larger than 400 bases.

For the first iteration, the already known ALFE sequences were used as queries. For the following iterations, the contigs selected from the previous iteration were used as queries. Due to the very large number of matches detected by BLASTN analysis after some iterations (repetitive regions from the flanking region of EFVEs), only 5 iterations were used in order to analyze the contigs obtained.

### Mass spectrometry.

C6/36 and Aag2 cells (10^7^) were lysed by sonication (twice for 20 s each time using an ultrasonic probe) in lysis buffer (urea, 6 M; Tris HCl, 150 mM, pH 8.8; β-octyl, 1%; dithiothreitol [DTT], 10 mM). In addition, in order to have an indication of the subcellular location of the proteins of interest, subcellular fractionation of the cell extracts was performed using a commercial kit (subcellular protein fractionation kit for cultured cells; Thermo Fisher Scientific) according to the manufacturer's instructions, resulting in 6 fractions: the cytoplasmic, membrane, soluble nuclear, chromatin-bound, cytoskeletal extract, and insoluble pellet fractions. Proteolysis was performed using a filter-assisted sample preparation strategy. Briefly, proteins were transferred over a filter with a 10-kDa-molecular-mass cutoff (Microcon, Amicon Merck), reduced, and alkylated (for DTT, 10 mM final concentration, 2 h at 37°C; for iodoacetamide, 50 mM final concentration, 30 min in the dark at room temperature). After the proteins were washed 3 times with ammonium bicarbonate (50 mM), the proteins were proteolysed with trypsin (10 ng modified sequencing-grade trypsin [Roche], 37°C, overnight). The resulting proteolytic peptides were recovered by centrifugation (15 min at 10,000 × *g*), acidified with 0.1% aqueous trifluoroacetic acid, and desalted using C_18_ sample preparation pipette tips (Ziptip C_18_; Millipore). The peptides were purified on a capillary reversed-phase column (C_18_ Acclaim PepMap; inside diameter, 75 μm; length, 50 cm; Thermo Fisher Scientific) at a constant flow rate of 220 nl/min with a gradient of 2% to 40% buffer B in buffer A over 170 min (buffer A, H_2_O, acetonitrile [ACN], and formic acid (FA) [98:2:0.1, vol/vol/vol]; buffer B, H_2_O, ACN, and FA [10:90:0.1, vol/vol/vol]). The MS analysis was performed on a Q Exactive mass spectrometer (Thermo Fisher Scientific) with a top 10 acquisition method (MS resolution, 70,000; mass range, 400 to 2,000 Da), followed by 1 MS/MS run on each of the 10 most intense peaks at a resolution of 17,500 with a dynamic exclusion for 90 s. Raw data were processed using Proteome Discoverer (v2.1) software (Thermo Fisher Scientific). The database search was done with the Mascot search engine (Matrix Science Mascot, v2.2.04) on a homemade protein data bank containing the putative proteins for endogenous viral elements as well as Aedes proteins (17,756 sequences). The following parameters were used: MS tolerance, 10 ppm; MS/MS tolerance, 0.02 Da; semitryptic peptides; two miscleavages allowed; partial modifications, carbamidomethylation (on cysteine), oxidation (on methionine), and deamidation (on asparagine and glutamine).

### Phylogenetic analysis of AEFE1.

DNA was extracted from individual A. aegypti mosquitoes. PCR was performed for the full-length AEFE1 element, and the amplicons were sequenced by the Sanger technique. Forward and reverse sequences were trimmed on the basis of the chromatogram quality and aligned to generate a consensus sequence using the program Geneious (v7) (52). Sequences from all successfully sequenced individuals, closely related ISFs, and medically important flaviviruses were aligned using the ClustalW program and trimmed to the same length. The program PHYML (53) was used to generate two phylogenetic trees using the PhyML best Akaike information criterion tree and the Fast likelihood-based method. The first tree contains closely related ISFs, medically relevant flaviviruses, and a representative AEFE1 sequence. The second tree was constructed using only the ISF sequences and the AEFE1 sequences from A. aegypti. The best nucleotide substitution method was general time reverse (GTR) +G for both trees.

### Accession number(s).

Sequences are available under NCBI BioProject numbers PRJNA386455 (Aedes aegypti transcriptome DENV1 versus the control at 24 and 96 hpi) and PRJNA386453 (Aedes aegypti transcriptome DENV1 versus DENV3).

## Supplementary Material

Supplemental material
