# Supplementary material for: Uncovering the Repertoire of Endogenous Flaviviral Elements in Aedes Mosquito Genomes
Source: J Virol. 2017 Jul 12;91(15):e00571-17. doi: 10.1128/JVI.00571-17 (PMC5512259; doi:10.1128/JVI.00571-17)
Supplement: Supplemental material [file supp_91_15_e00571-17__index.html]

Uncovering the Repertoire of Endogenous Flaviviral Elements in Aedes Mosquito Genomes — Supplemental material 

# Uncovering the Repertoire of Endogenous Flaviviral Elements in Aedes Mosquito Genomes

## Supplemental material

- Supplemental file 1 -

  Table S1 (List of proteins in C6/36 and Aag2 identified by MS analysis.)

  XLSX, 792K
